# Epilogue to a Metamorphosis

**DOI:** 10.1007/s11606-025-10134-3

**Published:** 2026-01-07

**Authors:** Tina Arkee

**Affiliations:** Vanderbilt University Medical Center, Nashville, TN USA

**Keywords:** Medical humanities, Poetry, End of life

*All that was lost and all that remained:*

Your life story as told through a loved one’s lens.

An inheritance of curiosity, perseverance, adventure –

The apple farm in Iowa

 becomes

The jungles of South America

 becomes

The walls of a university lecture hall

(And, somewhere in between)

A stint in the Pacific Islands

 becomes

A tour in Korea

 becomes

Long weeks as a prisoner of war

Your son, who followed in your footsteps

And took to the forest,

In search of the primordial yet evergreen,

Sits in silence with you, listening to the trees.

Your daughter, who holds your hand

When you cannot lift it yourself

*(Part of the self as a ghostly presence, a heaviness, a phantom)*

Reads to you from The Wind in the Willows,

A Christmas Eve tradition turned on its side,

Adapted for the circumstances,

Microevolution caught in the act.

Boundaries blur beyond recognition,

And then are crossed:

I skim through the archives of your past life

As a scholar-scientist,

Read obituaries for your dearly departed.

(And, later, on days away from the hospital, search for news of your passing)

To witness all of this, and then

To be asked about chances of recovery,

To redefine quality of life –

Two perspectives at odds with each other,

To consider the person as a whole, or

As a vessel for a single organ.

(And, later, a timeline for dying – *what to expect, and when to expect it*)

Long hours

become

Long days

become

More than a week of anticipation

In mostly silence, save for

The sounds of sheets rustling

And slowing breaths,

The sound of metamorphosis from man to mythology.

Finally, the end.

I awkwardly untangle my stethoscope from my hair

To listen for something that isn’t there,

In all its deafening absence.

Later, filling out paperwork, I wonder

How to distill nearly a century

Into a few words,

How to write an epilogue worthy of your story.

Author’s note:

I wrote this piece in the aftermath of a patient’s death. I’d met him 3 weeks earlier, when he was admitted to my inpatient medicine team under the diagnosis of “failure to thrive.”

I’d only spoken to him a couple of times before he lost the ability to communicate verbally. Despite this, I felt like I came to know who he was as a person over the course of his prolonged hospitalization, in part because a brief observation about a shared connection to the state of Iowa, where I did my MD/PhD, led to storytelling by his family members. The stories they told me painted a picture of someone who was very much thriving in their own way, someone who had lived an adventurous life, and who now found joy in the simplicities of life and brought joy to his caregivers.

After he took a turn for the worse, I worried about him when I was away from the hospital. When I was in the hospital, I spent time interpreting the comments of consulting teams and discussing the uncertainties of the dying process with his children. Eventually, I pronounced his death while his loved ones wept at his side. I felt strangely guilty for being affected by his passing when I’d only known him for a few weeks. How could my sadness compare to the grief that his family was experiencing? The brevity of our relationship made me feel like I hadn’t earned the right to mourn the loss of a man who had left a mark on generations of people, even as I’d come to learn about his life and values from those who loved him. It also made me feel guilty for inserting myself into his family’s grieving process. I’d expected myself to serve as a one-way mirror for my patient and his family, to hold space for them as they reminisced about their loved one and began to grieve his loss. Instead, I found myself struggling to separate my emotions from theirs, blurring boundaries once again.

I also wondered how the death pronouncement might have gone if I didn’t have a relationship with the family, how much more painful the process might have been for them if I was an unfamiliar face. The process of pronouncing death and completing the death certificate strips the emotion and humanity from the deeply intimate process of dying, reducing a life to a few facts without acknowledging who the deceased was. The physician has a responsibility to document as accurately as possible, but there is no real outlet for documenting an oral history of our patients. I wrote this poem in part because the death certificate and death summary did not provide the space to document who my patient was beyond the circumstances surrounding his demise, which made it difficult to find closure after such a powerful connection came to an end.

The relationship – however brief it may be – between patients (and their families) and physicians can be incredibly powerful. We meet them during periods of extreme vulnerability and uncertainty, and often don’t have satisfying answers for their questions, which can be frustrating for all parties involved. But we also have the opportunity to learn about who our patients are outside of the confines of their hospital rooms, and about the legacies that they hope to leave behind. This is a gift that I often find myself reflecting on.

## Declarations

### Privacy

All potentially identifiable details have been altered.

